# Personalized COPD Care: The Future of Precision-Based Therapies

**DOI:** 10.3390/jcm13216339

**Published:** 2024-10-23

**Authors:** Leslie K. Appleton, Nicola A. Hanania, Muhammad Adrish

**Affiliations:** Section of Pulmonary, Critical Care and Sleep Medicine, Baylor College of Medicine, 1504 Taub Loop, Houston, TX 77030, USAhanania@bcm.edu (N.A.H.)

**Keywords:** COPD, biologics, precision therapy

## Abstract

Chronic obstructive pulmonary disease (COPD) is a progressive respiratory illness characterized by long-standing respiratory symptoms and airflow limitation. It is a major contributor to respiratory disease-related deaths and currently ranked as the sixth leading cause of mortality in the United States. Approved pharmacological therapies for the stable disease primarily consist of inhaled short and long-acting bronchodilators, inhaled corticosteroids, azithromycin, and roflumilast. In recent years, significant progress has been made in the management of COPD through the identification of different COPD phenotypes and endotypes, which allows for a more personalized treatment approach. While earlier studies investigating targeted therapies were less promising, recent data on drugs targeting type 2 inflammatory pathways have shown promising results in carefully selected patients. In this article, we will review the available data on targeted therapies as well as the ongoing clinical studies of novel targeted therapies for COPD. Understanding and implementing these advancements hold promise for improving outcomes and quality of life for individuals living with COPD.

## 1. Introduction

Chronic obstructive pulmonary disease (COPD) is a progressive respiratory disease characterized by chronic symptoms of cough, dyspnea, and sputum production and is often complicated with recurrent exacerbations related to underlying chronic inflammation and subsequent progressive airflow limitation [1]. COPD is estimated to affect nearly 10% of the world’s population over age 40, and it remains one of the leading causes of death in both the United States and around the globe [2]. The true prevalence of COPD globally and by region is not well understood, largely due to underreporting or underdiagnosing as well as differences in data collection and criteria [1]. While some literature and analyses have emerged, they have been hyper-focused on specific countries or small regions rather than larger population-based studies. However, one recent modeling study currently estimates that as of 2020, North America had the highest prevalence of disease with nearly 17% of the population affected, followed closely by Sub-Saharan Africa at 15.5% and South America at 13.3%, then Europe and Central Asia at 10.3%, the Middle East at 9.4%, and East Asia at 8.6% [3]. This same study went on to project that the global prevalence of COPD would increase by 23% by 2050, with Sub-Saharan Africa becoming the leading region for COPD cases globally.

Per the Global Initiative for Chronic Obstructive Lung Disease (GOLD), the goals of COPD therapy are to reduce symptoms, improve lung function, exercise tolerance and quality of life, and reduce future risks of exacerbations and hospitalization as well as mortality [1]. Pharmacological therapy should be offered based on the presence of symptoms and risk of exacerbations, with a more nuanced lens towards the clinical phenotype. Several phenotypes have been identified including chronic bronchitis, emphysema, hyperinflation, asthma COPD overlap, bronchiectasis-COPD overlap, non-smoking COPD, frequent exacerbator, rapid decliner, pulmonary cachexia, and chronic respiratory failure. Multiple phenotypes may be present in a given patient [4,5]. Identification of these phenotypes can help providers identify patients who may respond to a specific intervention. For example, patients with chronic bronchitis may respond to phosphodiesterase-4 inhibitors, whereas patients with asthma–COPD overlap may have an increased response to inhaled corticosteroids [6]. The treatment for stable disease consists of a combination of inhaled and oral therapies, each of which has a specific indication for use. Inhaled medications include inhaled bronchodilators (long-acting muscarinic agent or LAMA and long-acting beta agonist or LABA) and inhaled corticosteroids (ICS) as stand alones or in combination; although, notably, bronchodilators may be less effective in patients who have more of an emphysematous phenotype [1]. ICS should be reserved for patients who have frequent or clinically significant exacerbations and evidence of type 2 inflammation [7,8,9]. Oral treatments (azithromycin and roflumilast) should be considered if the disease is not adequately controlled with inhaled therapies or in those with frequent exacerbations. However, despite the use of standard therapy including triple inhaled medications, studies suggest that up to one third of patients continue to have frequent or severe exacerbations [9,10].

In patients who remain unresponsive to the standard therapies, the identification of underlying molecular and pathophysiologic mechanisms that may be driving the disease process could help identify subgroups who might benefit from targeted therapies. Leveraging multi-omics techniques, these “treatable traits” have become the focus of recent research under the philosophy that certain complex diseases supersede their established disease archetypes and that specific biomarkers associated with disease may allow for more precise, targeted, and effective treatment strategies [11,12]. While certain genetic endotypes including alpha1-antitrypsin and telomerase polymorphisms have been well known for many years, recent research has been quite varied in the identification of newer endotypes. Some of the literature has focused on the airway microbiome as a contributor of disease, with numerous studies identifying specific pathogens that are distinct and carry higher risk for mortality in certain types of COPD. Other data have focused on the underlying host genetic predispositions that contribute to the airway microbiome in COPD patients [13,14,15,16]. Perhaps the best-studied endotypes are the ones expanding our knowledge of the underlying inflammatory mechanisms of COPD. These studies range from identifying specific cytokines associated with COPD exacerbations to identifying different inflammatory markers and pathways in patients with tuberculosis and biomass-related COPD, COPD-bronchiectasis patients, and COPD and asthma overlap patients, all of which present different options for intervention [17,18,19,20]. Of particular interest for this review is the emerging evidence regarding different inflammatory endotypes of COPD.

**Figure 1:**
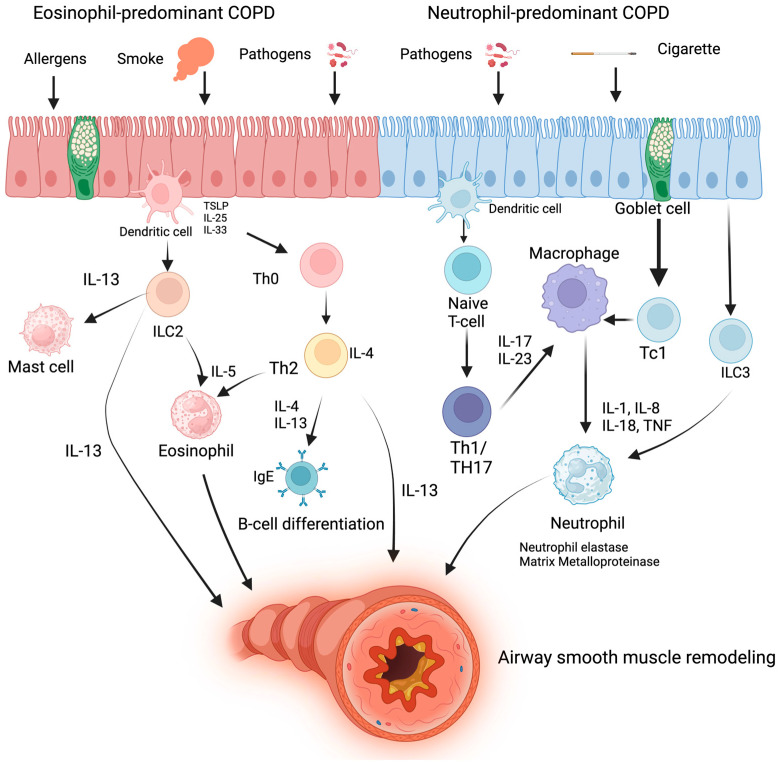
Inflammatory pathways in COPD.

For many years, the approach to COPD has taken the “the one size fits all” approach; however, it is now obvious that it is a heterogenous disorder, with inflammation being driven by both the innate and adaptive immune responses. Airway inflammation in COPD is also heterogenous with type 1 and type 3 inflammation characterized by neutrophilic airway inflammation, and type 2 inflammation characterized by eosinophilic inflammation. Most COPD patients have type 1 and 3 neutrophilic inflammation, which is thought to be triggered by a combination of cigarette smoke, oxidative stress which recruits neutrophils from the blood, and colonizing bacteria [21]. Proinflammatory cytokines such as IL-1, IL-6, and TNF-α are elevated in patients with COPD and lead to the activation of transcription factor nuclear factor (NF)-κB, which leads to inflammation. These patients are typically less responsive to ICS and may be at a higher risk of bacterial pneumonia [21]. However, newer evidence suggests that a significant proportion of patients have predominant type 2 (T2) inflammation. Signaling from alarmins such as thymic stromal lymphopoietin (TSLP), IL-25, and IL-33 promote this cycle mediated by the increased expression of proinflammatory cytokines including IL-4, IL-5, and IL-13 which are produced by T-helper cell type 2 (TH2) cells and group 2 innate lymphoid cells (ILC2) and characterized by elevated airway and blood eosinophils [22,23] (Figure 1). These changes result in the recruitment of eosinophils and other effector cells such as mast cells and basophils to the lungs, all of which contribute to airway remodeling, epithelial activation, increased mucus production, and IgE-induced allergen hypersensitivity. Outside of the lungs, T2 inflammation and eosinophils have also been linked to pathology throughout the airway and other organ systems including allergic rhinitis, atopic dermatitis, asthma, and nasal polyposis, suggesting that T2 inflammation is far more systemically pathologic beyond the lungs [24,25]. Increased peripheral blood eosinophils, which can be easily measured by a common blood test, have been used as a surrogate marker to detect the T2 inflammatory pathway and have been associated with ICS responsiveness [21]. While eosinophilic-mediated inflammation is more closely correlated with asthma, recent research not only identifies that many patients with COPD have this phenotype, but also identifies them as at higher risk for exacerbation [26]. Indeed, recent data show that 18–66% of patients with COPD have an eosinophilic phenotype [27,28]. Clinical trials of medications targeting cytokines within the T2 inflammatory pathway have been promising in asthma patients, with several studies either completed or currently underway in COPD patients [29]. Leveraging these endotyping strategies is the next step in precision medicine. It is in line with the newer, more broadly accepted approach to the “unified airway model”, in which there is interconnectedness between the pathophysiology of the upper and lower airways and the fact that the treatment of disease in one part of the airway is liable to be linked to pathology elsewhere in the airway and subsequently may confer a reduction in morbidity and mortality in high-risk COPD patients and beyond.

## 2. Targeted Therapies for COPD

### 2.1. Anti-IL-5/5Ra

Interleukin (IL) 5 is a cytokine in the β common chain family whose principal purpose is facilitating eosinophil differentiation in the bone marrow, maturation, mobilization, and recruitment at sites of inflammation, activation, and survival via the IL-5 receptor alpha (Rα) expressed on eosinophils [30,31]. In asthma patients, elevated levels of airway and blood eosinophils as well as IL-5 have been demonstrated in multiple studies and are considered to be a hallmark of certain asthma phenotypes, particularly those unresponsive or refractory to ICS, making them appealing therapeutic targets [31]. Monoclonal antibodies targeting IL-5 (mepolizumab, reslizumab) and IL-5Rα (benralizumab) were subsequently developed and have demonstrated the ability to reduce peripheral and sputum eosinophil counts, the number of exacerbations, the need for oral corticosteroids, and to improve the quality of life score in eosinophilic asthma patients [31,32]. Interestingly, a post hoc analysis of MENSA and DREAM trials also showed reduced rates of exacerbation in eosinophilic asthma patients with some overlapping clinical features with COPD [33].

Leveraging this knowledge, additional studies sought to determine the safety and efficacy of these drugs in COPD patients with an eosinophilic phenotype (Table 1).

In a randomized, double-blind, placebo-controlled multicenter phase II study comprised of 101 COPD patients, 100 milligrams (mg) of benralizumab were administered subcutaneously every four weeks for three doses, then every eight weeks for five doses in the treatment group. Benralizumab did not reduce the number of acute exacerbations and therefore did not meet the primary endpoint [34]; however, it did reduce the blood and sputum eosinophil counts and improved the forced expiratory volume in one second (FEV1), similar to asthma patients and particularly in those with higher blood eosinophil counts. Further phase III studies, namely, METREX and METREO for mepolizumab and GALATHEA and TERRANOVA for benralizumab, evaluated patients with COPD with the eosinophilic phenotype. In METREX and METREO, the eosinophilic phenotype was defined as ≥150/cubic millimeter (μL) at screening or ≥300/μL during the previous year. In METREX, patients received either 100 mg of mepolizumab or placebo every four weeks for 52 weeks. In METREO, patients were administered either 100 mg or 300 mg of mepolizumab or placebo every four weeks for 52 weeks. Both studies found a lower annual rate of moderate to severe exacerbations with 100 mg of mepolizumab, but the 300 mg dose failed to reach the statistical significance in METREO [35,36]. The subsequent phase III trial of mepolizumab (MATINEE) included COPD patients with blood eosinophils of ≥300/μL at screening. Recently, positive results of this study were announced with full publication awaited at the time of this writing [37].

**Table 1:** Targeted therapies in COPD.

Author	Trial Name	Drug Studied	Targeted Molecule	Year	Sample Size and Population	Primary Endpoint	Findings
Pavord, ID [35]	METREX NCT02105948	Mepolizumab	IL-5	2017	N = 837 with COPD and moderate to severe exacerbations while on triple therapy BEC ≥ 150 cells/μL at screening or ≥300 cells/μL during the past year	Annual rate of moderate or severe exacerbations	↓ rate of exacerbation 1.40 (rate ratio 0.82, 95% CI 0.68–0.98, *p* = 0.04) ↑ time to first moderate or severe exacerbation Safety profile similar to placebo
Pavord, ID [35]	METREO NCT02105961	Mepolizumab	IL-5	2017	N = 675, COPD and moderate to severe exacerbations while on triple therapy and BEC ≥ 150 cells/μL at screening or ≥300 cells/μL during the past year	Annual rate of moderate or severe exacerbations	↓ mean rate of exacerbation in treatment groups but not statistically significant No difference in rate of exacerbations between treatment groups (100 mg vs. 300 mg) Safety profile similar to placebo
Criner, GJ [38]	GALATHEA NCT02138916	Benralizumab	IL-5Rα	2019	N = 1120 with moderate to very severe COPD, COPD symptoms and ≥2 moderate or 1 severe exacerbation in the past year and with BEC ≥ 220 cells/μL	Annualized COPD exacerbation rate ratio at 56 weeks	Numerical but nonsignificant reduction in rate of annualized exacerbation Safety profile similar to placebo
Criner, GJ [38]	TERRANOVA NCT02155660	Benralizumab	IL-5Rα	2019	N = 1545 with moderate to very severe COPD, COPD symptoms and ≥2 moderate or 1 severe exacerbation in the past year and with BEC ≥ 220 cells/μL	Annualized COPD exacerbation rate ratio at 56 weeks	Numerical but nonsignificant reduction in rate of annualized exacerbation Safety profile similar to placebo
Bhatt, SP [39]	BOREAS NCT03930732	Dupilumab	IL-4Rα	2023	N = 939 COPD with BEC ≥300 cells/μL, FEV1/FVC <0.70, FEV1 30–70%, and on triple therapy with high risk of exacerbation	Annualized rate of moderate or severe COPD exacerbation	↓ rate of exacerbation 0.78 (95% CI 0.64–0.93) ↑ prebronchodilator FEV1 Adverse events similar to placebo
Bhatt, SP [40]	NOTUS NCT04456673	Dupilumab	IL-4Rα	2024	N = 935 with BEC ≥300 cells/μL, FEV1/FVC < 0.70, FEV1 30–70%, and on triple therapy with high risk of exacerbation	Annualized rate of moderate or severe exacerbation	↓ rate of exacerbation 0.86 (95% CI 0.70–1.06), rate ratio 0.66 (95% CI 0.54–0.82, *p* < 0.001) ↑ prebronchodilator FEV1 Adverse events similar to placebo
Yousuf, AJ [41]	COPD-ST2OP NCT03615040	Astegolimab	ST2 blocker	2022	N = 97 with moderate to very severe COPD and ≥2 exacerbations in the past year	Exacerbation rate with a negative binomial count model in the intention-to-treat population	No significant difference in exacerbation rate at 48 weeks ↑ patient reported SGRQ-C −3.3 (95% CI −6.4 to −0.2, *p* = 0.039) Treatment-emergent adverse events were similar between groups
Rabe, KF [42]	NCT03546907	Itepekimab	IL-33	2021	N = 343, aged 40–75 years, current or previous ≥10 pack year smoking history, diagnosis of COPD for at least 1 year	Annualized rate of moderate to severe exacerbation of COPD during 24–52-week treatment period	No significant change in annualized rate of exacerbation RR 0.81 (95%CI 0.61–1.07, *p* = 0.13) ↑ prebronchodilator FEV1 LSMD 0.06 (95% CI 0.01–0.10, *p* = 0.024) Treatment-emergent adverse events were similar between groups Nasopharyngitis (28 [16%] in the itepekimab group vs. 29 [17%] in the placebo group) Bronchitis (18 [10%] in itepekimab vs. 14 [8%] in placebo)
Singh, D [43]	NCT04039113	Tezepelumab	TSLP	2024	N = 333, patients aged 40–80 years, moderate to severe COPD	Annualized rate of moderate to severe exacerbation of COPD over 52 weeks	No statistically significant change in overall rate of moderate to severe exacerbation (17% relative reduction, *p* = 0.1042) Greater reduction observed in patients with blood eosinophils ≥ 300 cells/μL (37% reduction [95% CI 7, 57]) Adverse events and serious adverse events similar
NOVARTIS [44]	NCT00581945	Canakinumab	IL-1β	2010	N = 147 with COPD and FEV1 ≤ 50% and one exacerbation in the past 2 years	Change in FEV1, FVC, SVC, and FEF 25–75%	Numerical but nonsignificant reduction in exacerbation rate
Calverley, P [45]	NCT01448850	MEDI8968	IL-1 receptor 1 inhibitor	2017	N = 324, aged 45–75 years with ≥2 exacerbations in the past year	Moderate/severe acute exacerbations of COPD at week 56	No difference in exacerbation rate, lung function, or quality of life Incidence of treatment-emergent adverse events similar
Rennard, SI [46]		Infliximab	TNF-α	2007	N = 234, aged 40 years and older with moderate to severe COPD	Change from baseline in CRQ total score at week 24	No difference in CRQ Numerical increase in cancer and pneumonia
Rennard, SI [47]	REMICADE NCT00056264	Infliximab	TNF-α	2012	N = 234, who received ≥1 dose of infliximab for COPD	Number of patients with a malignancy and number of patients who died throughout the 5-year follow-up period	Cumulative incidence of malignancy over time was similar across all treatment groups Distribution of deaths across all groups was similar across groups
Lazaar, A [48]	NCT03034967	Danirixin	CXCR2 antagonist	2020	N = 614, aged 40–80 years with mild to moderate COPD	Incidence and severity of respiratory symptoms measured by E-RS: COPD scores	No difference in E-RS: COPD, SGRQ-C, or CAT scores Increased incidence of exacerbations and pneumonia in danirixin-treated groups

Abbreviations: BEC, blood eosinophil count; CAT, COPD Assessment Test; COPD, chronic obstructive pulmonary disease; CRQ, Chronic Respiratory Disease Questionnaire; E-RS: COPD, Evaluating Respiratory Symptoms in COPD; FEF 25–75, forced expiratory flow at 25 and 75% of the pulmonary volume; FEV1, forced expiratory volume in one second; FVC, forced vital capacity; N, number; SGRQ-C, St. George’s Respiratory Questionnaire for COPD patients.

GALATHEA and TERRANOVA included COPD patients with baseline blood eosinophil counts of ≥220/μL. In GALATHEA, patients received either 30 mg or 100 mg of benralizumab or placebo, whereas in TERRANOVA, patients were randomized to either 10 mg, 30 mg, or 100 mg of benralizumab or placebo. Patients received intervention every eight weeks for 56 weeks, respectively. Both of these studies failed to meet the primary endpoint of an annualized reduction in the rate of exacerbations when compared to placebo [38,49]. Post hoc analyses of GALATHEA and TERRANOVA identified a significant reduction in exacerbations in those receiving 100 mg benralizumab or those with certain clinical features such as frequent exacerbators, lower FEV1, and higher post-bronchodilator responses [50]. Ongoing work in the phase III RESOLUTE trial (NCT04053635) involves examining benralizumab in patients with moderate to very severe COPD plus a history of frequent exacerbations and a higher peripheral eosinophil count threshold of ≥300/μL [51]. These results are expected in the middle of the year 2025.

### 2.2. Anti-IL-4/IL-13

IL-4 and 13 (IL-4/IL-13) are well established cytokines associated with T-cell activity. While these are important parts of the host defense system against parasites, they are also responsible for a wide array of physiological inflammation. Both IL-4 and IL-13 are associated with a cluster of allergy-related genes and have been found to be upregulated in bronchial biopsies and sputum in asthma and allergic rhinitis patients [52]. In these populations, research demonstrates that IL-4 and IL-13 promote eosinophil recruitment and adhesion, increased mucus production, and fibrosis. IL-4 also plays an important role in B cell switching and IgE production. Dupilumab is a human monoclonal antibody designed against the IL-4 receptor alpha (Rα), which inhibits both IL-4 and IL-13 signaling. In steroid-dependent asthma patients, several studies have demonstrated a significant reduction in the rate of severe exacerbations as well as an improvement in FEV1 [53,54,55]. A post hoc analysis from LIBERTY ASTHMA QUEST examined the effect of dupilumab in asthma patients with persistent airway obstruction (defined as post-bronchodilator FEV1/FVC < 0.7) and found a significant improvement in lung function and reduction in severe exacerbations [56]. These findings raised interest about the potential role of dupilumab in a subgroup of patients with COPD with type 2 inflammation. The BOREAS trial was a large multicenter phase III trial of COPD patients treated with triple inhaled therapy with a baseline blood eosinophil count of ≥300/μL and high risk of exacerbation. Patients were randomized to receive either 300 mg dupilumab subcutaneously every two weeks or placebo for a total of 52 weeks. Dupilumab led to a 22% reduction in the annualized rate of moderate or severe exacerbations at 52 weeks. Additionally, FEV1 improved by about 83 mL by week 12, and the SGRQ scores were lower compared to the placebo [39]. More recently, data from the NOTUS trial, which used a similar dosing regimen and blood eosinophil count thresholds, showed a 34% reduction in the annualized rate of moderate or severe exacerbations compared to placebo [40]. Dupilumab also improved lung function and respiratory symptom scores, further confirming the proinflammatory role of IL-4 and IL-13 in these patients. In the dupilumab group, the IgE and fractional exhaled nitric oxide (FeNO) levels decreased during the 52-week study period. Dupilumab is the first biologic to be approved by the FDA for COPD patients [57].

### 2.3. Anti-Alarmins

There are three main alarmins, including IL-25, IL-33, and thymic stromal lymphopoietin (TSLP), which are produced by epithelial cells and play a role in regulating immune cell function in response to triggers. Together, these alarmins induce the release of cytokines belonging to the type 2 inflammatory cascade such as IL-5, IL-4, and IL-13 [58,59]. Of the three alarmins, IL-25 has no known human clinical trials for the treatment of COPD. Drugs targeting IL-33 and TSLP have been extensively studied in airway diseases and are summarized below.

IL-33 works by binding to the suppression of the tumorigenicity 2 (ST2) receptor and IL-1 receptor accessory protein (IL-1RAcP). This leads to activation of the transcription factor and proinflammatory kinases [60]. Drugs targeting the ST2 receptor have been used to block this signaling. Astegolimab, which is an ST2 blocker, was studied in a phase IIa, randomized, double-blind study of patients with moderate to severe COPD [41]. While there was no significant difference in the rate of exacerbation, patients had an improvement in health status as measured by Saint George’s Respiratory Questionnaire for COPD (SGRQ-C). Further phase IIb and phase III studies of astegolimab in COPD are ongoing [61,62]. Itepekimab is a monoclonal antibody that targets IL-33 and has demonstrated activity in patients with moderate to severe asthma [63]. In a proof-of-concept study, Rabe and colleagues tested whether genetic variants in the IL-33 pathway are associated with COPD [42]. In this two-part study, a genetic analysis followed by a phase IIa double-blind, randomized, placebo-controlled study were performed. The study demonstrated the association of gain of function and IL1RL1 variants with an increased risk of COPD and loss of function in IL-33 with a reduced risk of COPD. While there were no differences in exacerbation risks in the overall population, the subgroup analysis showed that exacerbations were lower, and lung function improved in former smokers. Multiple phase III studies assessing the long-term safety and tolerability of itepekimab (AERIFY-4) in COPD are ongoing [64,65,66]. Tozorakimab is a human monoclonal antibody that inhibits both reduced and oxidized IL-33 activities via a distinct ST2 and receptor for advanced glycation end products/epidermal growth factor receptor (RAGE/EGFR complex) signaling pathways [67]. In a phase I study of COPD patients, tozorakimab significantly reduced serum IL-5 and IL-13 compared to placebo [68]. A phase II study is evaluating the safety, efficacy, and tolerability of tozorakimab in patients with moderate to severe COPD [69].

Tezepelumab is a monoclonal antibody targeting TSLP and has been known to have beneficial effects in patients with severe, uncontrolled asthma [70]. Recently, data from a phase IIa double-blind COURSE study (NCT04039113) were presented [43]. There was a numerical reduction in the overall rate of moderate to severe exacerbations (17% relative reduction, *p* = 0.1042), but statistical significance was not reached. Greater reductions in exacerbations were observed in patients with blood eosinophils ≥300 cells/μL (37% reduction [95% CI 7, 57]). There were also trends of improvement in pre-bronchodilator FEV1 and SGRQ with Tezepelumab use. These findings will likely pave the way for future phase III studies.

### 2.4. Drugs Targeting Neutrophilic Inflammation

IL-1 is a proinflammatory cytokine, which is produced by stromal and various immune cells, and is thought to play a role in COPD pathogenesis [71]. IL-1α and IL-1β are elevated in patients with stable COPD as well as during the exacerbation phase of COPD and have been linked to the neutrophilic inflammation seen in response to cigarette smoke [72]. Canakinumanb is a monoclonal antibody against IL-1β, which did not reach its primary endpoint in a study on COPD patients [44]. MEDI8968 is an antibody that binds to the IL-1 receptor inhibitor and inhibits its activation by IL-1α and IL-1β. In a phase II randomized, double-blind, placebo-controlled trial of COPD patients, there was no difference in the exacerbation rate, lung function, or health status outcomes with this drug [45]. The authors of the study suggested that patients with COPD and a high neutrophil count may benefit from such therapy; however, the absence of suitable biomarkers will make it difficult to identify this subpopulation.

TNF- α is a cytokine that has been implicated in the pathogenesis of COPD. It has been shown to impair the removal of apoptotic cells from lungs, which leads to a release of proinflammatory mediators [73]. Infliximab is a monoclonal antibody that binds to soluble and membrane-bound TNF-α and blocks in activity. In a pilot study of sixteen cachectic patients with moderate to severe COPD, infliximab did not produce an observable decrease in local inflammation [74]. In a small phase II study of 22 patients with mild to moderate COPD, no clinically beneficial effects were seen with infliximab [75]. A larger phase II study using infliximab in moderate to severe COPD patients also failed to show a benefit and instead noted a numerical increase in cancer and pneumonia incidence in infliximab-treated patients [46]. A subsequent study collected malignancy and mortality data from the completed infliximab studies in COPD for 5 years and noted that the increased incidence of malignancies noted in the phase II study diminished over time [47]. Etanercept is another drug which blocks TNF-α [76]. In a study of 81 patients with acute COPD exacerbation, etanercept was compared with prednisone and was found to have similar efficacy in improving FEV1 [76].

IL-8 has been found to be elevated in the sputum of COPD patients and plays a role in neutrophil chemoattraction. In a randomized, double-blind, placebo-controlled trial of 109 patients with stable COPD, an anti-IL-8 antibody, ABX-IL8, was compared with placebo [77]. There was improvement in the dyspnea index with ABX-IL8 compared to placebo, but no change was observed in the lung function, 6 min walk distance, and health status between the two groups.

Dysregulated IL-17 has been implicated in the pathogenesis of neutrophil-predominant COPD. CNTO6785 is an anti-IL17A monoclonal antibody that was studied in patients with symptomatic moderate to severe COPD in a phase II clinical trial [78]. Compared with placebo, there was an improvement in pre-bronchodilator FEV1 at week 16. Overall, adverse events were similar between the two groups.

CXC chemokine receptor 2 (CXCR2) antagonists have been known to block neutrophil migration and activation. In a phase IIb placebo-controlled study, a CXCR2 antagonist, danirixin, was administered in an escalating dose to patients with mild to moderate COPD [48]. The authors noted that there was an increase in the incidence of exacerbation and pneumonia in participants treated with danirixin. MK-7123 is another CXCR2 antagonist that was studied in a phase II proof-of-concept study in patients with moderate to severe COPD [79]. MK-7123 improved FEV1 compared to placebo; however, there were dose-related discontinuations due to a decrease in absolute neutrophil counts.

## 3. Conclusions

Airway inflammation in COPD is complex, involving a variety of inflammatory cells, including neutrophils, macrophages, eosinophils, mast cells, and CD8+ lymphocytes. These cells play significant roles in the disease’s inflammatory processes. Recent studies targeting type 2 inflammation in a subset of COPD patients have offered new hope for treatment. Dupilumab is the first biologic to be approved by the FDA for the treatment of eosinophilic COPD. More recently, positive results were also announced from the phase III study of mepolizumab in COPD patients. There were some encouraging results from earlier studies of benralizumab, tezepelumab, itepekimab, tozorakimab, and astegolimab, all of which are being studied in phase III trials with data expected in the coming years. Data on the use of precision therapies in neutrophil-predominant COPD are less promising, where the mainstay of treatment includes long-acting bronchodilators, macrolides, and phosphodiesterase-4 inhibitors. As research in the field of COPD progresses, we are developing a better understanding of the interactions between inflammatory endotypes and clinical phenotypes that will move the management from precision medicine to an individualized approach for each patient. Future research should also focus on therapies that have the potential to reverse or cause remission of COPD, offering a more definitive solution for patients.

## Data Availability

No new data were created for this study.

## References

[1] Global Initiative for Chronic Obstructive Lung Disease (GOLD) Global Strategy for the Diagnosis, Management and Prevention of Chronic Obstructive Pulmonary Disease: 2024 Report. https://www.goldcopd.org.

[2] Kochanek K.D., Murphy S.L., Xu J., Arias E. Mortality in the United States, 2016. NCHS Data Brief 2017; 293. https://www.cdc.gov/nchs/data/databriefs/db293.pdf.

[3] Boers E., Barrett M., Su J.G., Benjafield A.V., Sinha S., Kaye L., Zar H.J., Vuong V., Tellez D., Gondalia R. (2023). Global Burden of Chronic Obstructive Pulmonary Disease Through 2050. JAMA Netw. Open.

[4] Corlateanu A., Mendez Y., Wang Y., Garnica R.J.A., Botnaru V., Siafakas N. (2020). Chronic obstructive pulmonary disease and phenotypes: A state-of-the-art. Pulmonology.

[5] Barnes P.J. (2019). Inflammatory endotypes in COPD. Allergy.

[6] Miravitlles M., Soler-Cataluña J.J., Calle M., Soriano J.B. (2013). Treatment of COPD by clinical phenotypes: Putting old evidence into clinical practice. Eur. Respir. J..

[7] Pascoe S., Locantore N., Dransfield M.T., Barnes N.C., Pavord I.D. (2015). Blood eosinophil counts, exacerbations, and response to the addition of inhaled fluticasone furoate to vilanterol in patients with chronic obstructive pulmonary disease: A sec-ondary analysis of data from two parallel randomised controlled trials. Lancet Respir. Med..

[8] Bafadhel M., McKenna S., Terry S., Mistry V., Pancholi M., Venge P., Lomas D.A., Barer M.R., Johnston S.L., Pavord I.D. (2012). Blood eosinophils to direct corticosteroid treatment of exacerbations of chronic obstructive pulmonary disease: A randomized placebo-controlled trial. Am. J. Respir. Crit. Care Med..

[9] Vestbo J., Papi A., Corradi M., Blazhko V., Montagna I., Francisco C., Cohuet G., Vezzoli S., Scuri M., Singh D. (2017). Single inhaler extrafine triple therapy versus long-acting muscarinic antagonist thera-py for chronic obstructive pulmonary disease (TRINITY): A double-blind, parallel group, randomised controlled trial. Lancet.

[10] Halpin D.M.G., Dransfield M.T., Han M.K., Jones C.E., Kilbride S., Lange P., Lipson D.A., Lomas D.A., Martinez F.J., Pascoe S. (2020). The effect of exacerbation history on outcomes in the IMPACT trial. Eur. Respir. J..

[11] Cardoso J., Ferreira A.J., Guimarães M., Oliveira A.S., Simão P., Sucena M. (2021). Treatable Traits in COPD—A Proposed Approach. Int. J. Chronic Obstr. Pulm. Dis..

[12] Polverino F., Sin D.D. (2024). Type 2 airway inflammation in COPD. Eur. Respir. J..

[13] Wang Z., Locantore N., Haldar K., Ramsheh M.Y., Beech A.S., Ma W., Brown J.R., Tal-Singer R., Barer M.R., Bafadhel M. (2021). Inflammatory Endotype-associated Airway Microbiome in Chronic Obstructive Pulmonary Disease Clinical Stability and Exacerbations: A Multicohort Longitudinal Analysis. Am. J. Respir. Crit. Care Med..

[14] Mayhew D., Devos N., Lambert C., Brown J.R., Clarke S.C., Kim V.L., Magid-Slav M., E Miller B., Ostridge K.K., Patel R. (2018). Longitudinal profiling of the lung microbiome in the AERIS study demonstrates repeatability of bacterial and eosinophilic COPD exacerbations. Thorax.

[15] Dicker A.J., Huang J.T.J., Lonergan M., Keir H.R., Fong C.J., Tan B., Cassidy A.J., Finch S., Mullerova H., Miller B.E. (2021). The sputum microbiome, airway inflammation, and mortality in chronic ob-structive pulmonary disease. J. Allergy Clin. Immunol..

[16] Gao J., Yang Y., Xiang X., Zheng H., Yi X., Wang F., Liang Z., Chen D., Shi W., Wang L. (2024). Human genetic associations of the airway microbiome in chronic obstructive pulmonary disease. Respir. Res..

[17] Bade G., Khan M.A., Srivastava A.K., Khare P., Solaiappan K., Guleria R., Palaniyar N., Talwar A. (2014). Serum cytokine profiling and enrichment analysis reveal the involvement of immunological and inflammatory pathways in stable patients with chronic obstructive pulmonary disease. Int. J. Chronic Obstr. Pulm. Dis..

[18] Siddharthan T., Gupte A., Barnes P.J. (2020). Chronic Obstructive Pulmonary Disease Endotypes in Low- and Middle-Income Country Settings: Precision Medicine for All. Am. J. Respir. Crit. Care Med..

[19] Huang J.T., Cant E., Keir H.R., Barton A.K., Kuzmanova E., Shuttleworth M., Pollock J., Finch S., Polverino E., Bottier M. (2022). Endotyping Chronic Obstructive Pulmonary Disease, Bronchiectasis, and the “Chronic Obstructive Pulmonary Disease-Bronchiectasis Association”. Am. J. Respir. Crit. Care Med..

[20] Bhatt S.P., Agusti A., Bafadhel M., Christenson S.A., Bon J., Donaldson G.C., Sin D.D., Wedzicha J.A., Martinez F.J. (2023). Phenotypes, Etiotypes, and Endotypes of Exacerbations of Chronic Obstructive Pulmonary Disease. Am. J. Respir. Crit. Care Med..

[21] Barnes P.J. (2021). Endo-phenotyping of COPD patients. Expert. Rev. Respir. Med..

[22] Barnes P.J. (2009). The cytokine network in chronic obstructive pulmonary disease. Am. J. Respir. Cell Mol. Biol..

[23] McGregor M.C., Krings J.G., Nair P., Castro M. (2019). Role of Biologics in Asthma. Am. J. Respir. Crit. Care Med..

[24] Giombi F., Pace G.M., Pirola F., Cerasuolo M., Ferreli F., Mercante G., Spriano G., Canonica G.W., Heffler E., Ferri S. (2024). Airways Type-2 Related Disorders: Multiorgan, Systemic or Syndemic Disease?. Int. J. Mol. Sci..

[25] Giavina-Bianchi P., Aun M.V., Takejima P., Kalil J., Agondi R.C. (2016). United airway disease: Current perspectives. J. Asthma Allergy.

[26] Bafadhel M., McKenna S., Terry S., Mistry V., Reid C., Haldar P., McCormick M., Haldar K., Kebadze T., Duvoix A. (2011). Acute exacerbations of chronic obstructive pulmonary disease: Identification of biologic clusters and their biomarkers. Am. J. Respir. Crit. Care Med..

[27] Singh D., Kolsum U., Brightling C.E., Locantore N., Agusti A., Tal-Singer R. (2014). Eosinophilic inflammation in COPD: Prevalence and clinical characteristics. Eur. Respir. J..

[28] Wu H.X., Zhuo K.Q., Cheng D.Y. (2019). Prevalence and Baseline Clinical Characteristics of Eosinophilic Chronic Obstructive Pul-monary Disease: A Meta-Analysis and Systematic Review. Front. Med..

[29] Kersul A.L., Cosio B.G. (2024). Biologics in COPD. Open Respir. Arch..

[30] Kolbeck R., Kozhich A., Koike M., Peng L., Andersson C.K., Damschroder M.M., Reed J.L., Woods R., Dall’Acqua W.W., Stephens G.L. (2010). MEDI-563, a humanized anti-IL-5 receptor alpha mAb with enhanced anti-body-dependent cell-mediated cytotoxicity function. J. Allergy Clin. Immunol..

[31] Varricchi G., Bagnasco D., Borriello F., Heffler E., Canonica G.W. (2016). Interleukin-5 pathway inhibition in the treatment of eo-sinophilic respiratory disorders: Evidence and unmet needs. Curr. Opin. Allergy Clin. Immunol..

[32] Ortega H.G., Liu M.C., Pavord I.D., Brusselle G.G., FitzGerald J.M., Chetta A., Humbert M., Katz L.E., Keene O.N., Yancey S.W. (2014). Mepolizumab treatment in patients with severe eosinophilic asthma. N. Engl. J. Med..

[33] Pavord I.D., Bel E.H., Bourdin A., Chan R., Han J.K., Keene O.N., Liu M.C., Martin N., Papi A., Roufosse F. (2022). From DREAM to REALITI-A and beyond: Mepolizumab for the treatment of eosin-ophil-driven diseases. Allergy.

[34] Brightling C.E., Bleecker E.R., Panettieri R.A., Bafadhel M., She D., Ward C.K., Xu X., Birrell C., van der Merwe R. (2014). Benralizumab for chronic obstructive pulmonary disease and sputum eosinophilia: A randomised, double-blind, placebo-controlled, phase 2a study. Lancet Respir. Med..

[35] Pavord I.D., Chanez P., Criner G.J., Kerstjens H.A., Korn S., Lugogo N., Martinot J.-B., Sagara H., Albers F.C., Bradford E.S. (2017). Mepolizumab for Eosinophilic Chronic Obstructive Pulmonary Disease. N. Engl. J. Med..

[36] Pavord I.D., Chapman K.R., Bafadhel M., Sciurba F.C., Bradford E.S., Harris S.S., Mayer B., Rubin D.B., Yancey S.W., Paggiaro P. (2021). Mepolizumab for Eosinophil-Associated COPD: Analysis of METREX and METREO. Int. J. Chron. Obs. Pulmon. Dis..

[37] GSK Announces Positive Results from Phase III Trial of Nucala (Mepolizumab) in COPD. GSK. https://www.gsk.com/en-gb/media/press-releases/gsk-announces-positive-results-from-phase-iii-trial-of-nucala-mepolizumab-in-copd/.

[38] Criner G.J., Celli B.R., Brightling C.E., Agusti A., Papi A., Singh D., Sin D.D., Vogelmeier C.F., Sciurba F.C., Bafadhel M. (2019). Benralizumab for the Prevention of COPD Exacerbations. N. Engl. J. Med..

[39] Bhatt S.P., Rabe K.F., Hanania N.A., Vogelmeier C.F., Cole J., Bafadhel M., Christenson S.A., Papi A., Singh D., Laws E. (2023). Dupilumab for COPD with Type 2 Inflammation Indicated by Eosinophil Counts. N. Engl. J. Med..

[40] Bhatt S.P., Rabe K.F., Hanania N.A., Vogelmeier C.F., Bafadhel M., Christenson S.A., Papi A., Singh D., Laws E., Patel N. (2024). Dupilumab for COPD with Blood Eosinophil Evidence of Type 2 Inflammation. N. Engl. J. Med..

[41] Yousuf A.J., Mohammed S., Carr L., Ramsheh M.Y., Micieli C., Mistry V., Haldar K., Wright A., Novotny P., Parker S. (2022). Astegolimab, an anti-ST2, in chronic obstructive pulmonary disease (COPD-ST2OP): A phase 2a, placebo-controlled trial. Lancet Respir. Med..

[42] Rabe K.F., Celli B.R., Wechsler M.E., Abdulai R.M., Luo X., Boomsma M.M., Staudinger H., Horowitz J.E., Baras A., Ferreira M.A. (2021). Safety and efficacy of itepekimab in patients with moderate-to-severe COPD: A genetic association study and randomised, double-blind, phase 2a trial. Lancet Respir. Med..

[43] Singh D., Bafadhel M., Brightling C.E., Rabe K.F., Han M.K., Bhutani M., Bourbeau J., Christenson S.A., Dransfield M.T., Nair P. (2024). Tezepelumab in Adults with Moderate to Very Severe Chronic Obstructive Pulmonary Disease (COPD): Efficacy and Safety From the Phase 2a COURSE Study (abstract). Am. J. Respir. Crit. Care Med..

[44] Novartis (2007). Safety and Efficacy of Multiple Doses of Canakinumab (ACZ885) in Chronic Obstructive Pulmonary Disease (COPD) Patients. https://clinicaltrials.gov/ct2/show/NCT00581945.

[45] Calverley P.M.A., Sethi S., Dawson M., Ward C.K., Finch D.K., Penney M., Newbold P., van der Merwe R. (2017). A randomised, placebo-controlled trial of anti-interleukin-1 receptor 1 mono-clonal antibody MEDI8968 in chronic obstructive pulmonary disease. Respir. Res..

[46] Rennard S.I., Fogarty C., Kelsen S., Long W., Ramsdell J., Allison J., Mahler D., Saadeh C., Siler T., Snell P. (2007). The safety and efficacy of infliximab in moderate to severe chronic obstructive pulmonary disease. Am. J. Respir. Crit. Care Med..

[47] Rennard S.I., Flavin S.K., Agarwal P.K., Lo K.H., Barnathan E.S. (2013). Long-term safety study of infliximab in moderate-to-severe chronic obstructive pulmonary disease. Respir. Med..

[48] Lazaar A.L., Miller B.E., Donald A.C., Keeley T., Ambery C., Russell J., Watz H., Tal-Singer R. (2020). CXCR2 antagonist for patients with chronic obstructive pulmonary disease with chronic mucus hypersecretion: A phase 2b trial. Respir. Res..

[49] Mkorombindo T., Dransfield M.T. (2019). Mepolizumab in the treatment of eosinophilic chronic obstructive pulmonary disease. Int. J. Chron. Obs. Pulmon. Dis..

[50] Criner G.J., Celli B.R., Singh D., Agusti A., Papi A., Jison M., Makulova N., Shih V.H., Brooks L., Barker P. (2020). Predicting response to benralizumab in chronic obstructive pulmonary disease: Anal-yses of GALATHEA and TERRANOVA studies. Lancet Respir. Med..

[51] A Study to Evaluate Efficacy, Safety, Tolerability, and Pharmacokinetics of MEDI3506 in Participants with Moderate to Severe Chronic Obstructive Pulmonary Disease. ClinicalTrials.gov. https://clinicaltrials.gov/study/NCT04053634.

[52] May R.D., Fung M. (2015). Strategies targeting the IL-4/IL-13 axes in disease. Cytokine.

[53] Rabe K.F., Nair P., Brusselle G., Maspero J.F., Castro M., Sher L., Zhu H., Hamilton J.D., Swanson B.N., Khan A. (2018). Efficacy and Safety of Dupilumab in Glucocorticoid-Dependent Severe Asthma. N. Engl. J. Med..

[54] Castro M., Corren J., Pavord I.D., Maspero J., Wenzel S., Rabe K.F., Busse W.W., Ford L., Sher L., Fitzgerald J.M. (2018). Dupilumab Efficacy and Safety in Moderate-to-Severe Uncontrolled Asthma. N. Engl. J. Med..

[55] Wenzel S., Castro M., Corren J., Maspero J., Wang L., Zhang B., Pirozzi G., Sutherland E.R., Evans R.R., Joish V.N. (2016). Dupilumab efficacy and safety in adults with uncontrolled persistent asthma despite use of medium-to-high-dose inhaled corticosteroids plus a long-acting β2 agonist: A randomised double-blind place-bo-controlled pivotal phase 2b dose-ranging trial. Lancet.

[56] Hanania N.A., Castro M., Bateman E., Pavord I.D., Papi A., FitzGerald J.M., Maspero J.F., Katelaris C.H., Singh D., Daizadeh N. (2023). Efficacy of dupilumab in patients with moderate-to-severe asthma and persis-tent airflow obstruction. Ann. Allergy Asthma Immunol..

[57] Sanofi Announces Positive Phase 3 Trial Results for Dupixent^®^ (dupilumab) in Chronic Obstructive Pulmonary Disease (COPD). Sanofi. https://www.sanofi.com/en/media-room/press-releases/2024/2024-09-27-13-35-00-2954551.

[58] Deng C., Peng N., Tang Y., Yu N., Wang C., Cai X., Zhang L., Hu D., Ciccia F., Lu L. (2021). Roles of IL-25 in Type 2 Inflammation and Autoimmune Pathogene-sis. Front. Immunol..

[59] Porsbjerg C.M., Sverrild A., Lloyd C.M., Menzies-Gow A.N., Bel E.H. (2020). Anti-alarmins in asthma: Targeting the airway ep-ithelium with next-generation biologics. Eur. Respir. J..

[60] Pelaia C., Pelaia G., Longhini F., Crimi C., Calabrese C., Gallelli L., Sciacqua A., Vatrella A. (2021). Monoclonal Antibodies Targeting Alarmins: A New Perspective for Biological Therapies of Severe Asthma. Biomedicines.

[61] A Study to Investigate the Efficacy and Safety of Astegolimab in Participants with Chronic Obstructive Pulmonary Dis-ease (COPD). ClinicalTrials.gov. https://clinicaltrials.gov/study/NCT05878769?cond=copd&intr=Astegolimab&rank=1.

[62] A Study to Investigate the Efficacy and Safety of Astegolimab in Participants with COPD and Eosinophilic Inflamma-tion. ClinicalTrials.gov. https://clinicaltrials.gov/study/NCT05595642?cond=copd&intr=Astegolimab&rank=2.

[63] Wechsler M.E., Ruddy M.K., Pavord I.D., Israel E., Rabe K.F., Ford L.B., Maspero J.F., Abdulai R.M., Hu C.-C., Martincova R. (2021). Efficacy and Safety of Itepekimab in Patients with Moderate-to-Severe Asth-ma. N. Engl. J. Med..

[64] A Study to Evaluate the Efficacy and Safety of Itepekimab in Participants with Chronic Obstructive Pulmonary Disease (COPD). ClinicalTrials.gov. https://clinicaltrials.gov/study/NCT06208306.

[65] Study to Assess the Efficacy, Safety, and Tolerability of SAR440340/REGN3500/Itepekimab in Chronic Obstructive Pul-monary Disease (COPD) (AERIFY-1). ClinicalTrials.gov. https://clinicaltrials.gov/study/NCT04701983.

[66] (AERIFY-2). ClinicalTrials.gov. https://clinicaltrials.gov/study/NCT04751487.

[67] England E., Rees D.G., Scott I.C., Carmen S., Chan D.T.Y., Huntington C.E.C., Houslay K.F., Erngren T., Penney M., Majithiya J.B. (2023). Tozorakimab (MEDI3506): An anti-IL-33 antibody that inhibits IL-33 signalling via ST2 and RAGE/EGFR to reduce inflammation and epithelial dysfunction. Sci. Rep..

[68] Reid F., Singh D., Albayaty M., Moate R., Jimenez E., Sadiq M.W., Howe D., Gavala M., Killick H., Williams A. (2024). A Randomized Phase I Study of the Anti-Interleukin-33 Antibody Tozorakimab in Healthy Adults and Patients with Chronic Obstructive Pulmonary Disease. Clin. Pharmacol. Ther..

[69] A Phase, I.I. Randomized, Double-Blind, Placebo-Controlled Study to Assess MEDI3506 in Participants with COPD and Chronic Bronchitis (FRONTIER-4). ClinicalTrials.gov. https://clinicaltrials.gov/study/NCT04631016?rank=1.

[70] Menzies-Gow A., Corren J., Bourdin A., Chupp G., Israel E., Wechsler M.E., Brightling C.E., Griffiths J.M., Hellqvist Å., Bowen K. (2021). Tezepelumab in Adults and Adolescents with Severe, Uncontrolled Asthma. N. Engl. J. Med..

[71] Caramori G., Adcock I.M., Di Stefano A., Chung K.F. (2014). Cytokine inhibition in the treatment of COPD. Int. J. Chron. Obstruct Pulmon Dis..

[72] Botelho F.M., Bauer C.M.T., Finch D., Nikota J.K., Zavitz C.C.J., Kelly A., Lambert K.N., Piper S., Foster M.L., Goldring J.J.P. (2011). IL-1alpha/IL-1R1 expression in chronic obstructive pulmonary disease and mechanistic relevance to smoke-induced neutrophilia in mice. PLoS ONE.

[73] Borges V.M., Vandivier R.W., McPhillips K.A., Kench J.A., Morimoto K., Groshong S.D., Richens T.R., Graham B.B., Muldrow A.M., Van Heule L. (2009). TNFα inhibits apoptotic cell clearance in the lung, exacerbating acute inflammation. Am. J. Physiol. Lung Cell Mol. Physiol..

[74] Dentener M.A., Creutzberg E.C., Pennings H.J., Rijkers G.T., Mercken E., Wouters E.F. (2008). Effect of infliximab on local and systemic inflammation in chronic obstructive pulmonary disease: A pilot study. Respiration.

[75] van der Vaart H., Koëter G.H., Postma D.S., Kauffman H.F., ten Hacken N.H. (2005). First study of infliximab treatment in patients with chronic obstructive pulmonary disease. Am. J. Respir. Crit. Care Med..

[76] Aaron S.D., Vandemheen K.L., Maltais F., Field S.K., Sin D.D., Bourbeau J., Marciniuk D.D., FitzGerald J.M., Nair P., Mallick R. (2013). TNFα antagonists for acute exacerbations of COPD: A randomised double-blind controlled trial. Thorax.

[77] Mahler D.A., Huang S., Tabrizi M., Bell G.M. (2004). Efficacy and safety of a monoclonal antibody recognizing interleukin-8 in COPD: A pilot study. Chest.

[78] Eich A., Urban V., Jutel M., Vlcek J., Shim J.J., Trofimov V.I., Liam C.-K., Kuo P.-H., Hou Y., Xiao J. (2017). A Randomized, Placebo-Controlled Phase 2 Trial of CNTO 6785 in Chronic Obstructive Pulmonary Disease. COPD J. Chronic Obstr. Pulm. Dis..

[79] Rennard S.I., Dale D.C., Donohue J.F., Kanniess F., Magnussen H., Sutherland E.R., Watz H., Lu S., Stryszak P., Rosenberg E. (2015). CXCR2 Antagonist MK-7123. A Phase 2 Proof-of-Concept Trial for Chronic Obstructive Pulmonary Disease. Am. J. Respir. Crit. Care Med..

